# Comprehensive Long‐Term Orthodontic Management for an Adolescent Patient With Compromised First Permanent Molars

**DOI:** 10.1002/ccr3.73031

**Published:** 2026-06-30

**Authors:** Yen Tran, Desmond Cheer‐Vern Ong

**Affiliations:** ^1^ University of Otago Dunedin New Zealand; ^2^ Discipline of Orthodontics, School of Dentistry University of Queensland Brisbane Queensland Australia; ^3^ Private Practice Townsville Australia

**Keywords:** compromised first permanent molars, orthodontic treatment, third molar eruption

## Abstract

Comprehensive orthodontic treatment can provide excellent long‐term oral health outcomes for adolescent patients with compromised first permanent molars. Eliminating impending restorative requirements, resolving the malocclusion, and improving the likelihood of future third molar eruption are all potential advantages of this treatment approach.

## 1 Introduction

First permanent molars are commonly affected by structural compromises, which may result from dental caries [1, 2, 3], hypomineralization [4], and the deterioration of pre‐existing restorations [5]. Such compromises may result in various clinical dilemmas.

In scenarios where compromised first permanent molars are justifiably considered for removal, comprehensive orthodontic treatment is likely to be extremely valuable, if such treatment is accessible. The presenting occlusal scheme and the severity of intra‐arch crowding must be thoroughly assessed with consideration given to the horizontal and vertical skeletal morphology, as these factors may affect the techniques used, along with the difficulty or predictability of extraction space closure [6, 7, 8]. The presence, position, and developmental stage of the second and third permanent molars are also important considerations when formulating the most appropriate clinical management strategy [9, 10].

Adolescent patients who present with compromised first permanent molars often pose a multitude of clinical dilemmas. The central issue revolves around the probable time discrepancy between the life expectancy of the compromised molar and the patient's actual life expectancy, along with the cost–benefit perspective of the treatment option chosen. Should the decision be made to remove one or more compromised first permanent molars, a comprehensive orthodontic assessment is strongly recommended to ensure that the timing of any extraction is tailored to the presenting malocclusion and the option for subsequent orthodontic treatment is carefully planned to ensure the best possible treatment outcome [6].

Should a compromised first permanent molar be removed, the treatment objective is usually to have the adjacent second permanent molar erupt into or alternatively be orthodontically positioned into satisfactory contact with the second premolar tooth [6, 7]. If present, a third molar can serve as a functional replacement for a compromised permanent molar in that respective quadrant.

## 2 Case Presentation

A medically healthy 12‐year‐old patient in the late mixed dentition stage of dental development presented for an orthodontic consultation. A Class I malocclusion with an anterior overjet of 2.5 mm and 25% vertical anterior overbite was noted (Figure 1). The mandibular dental midline was located 1 mm to the left side of the maxillary dental midline and the facial midline. The maxillary right first permanent molar (16), the mandibular left first permanent molar (36), and the mandibular right second primary molar (75) had been restored with stainless‐steel crowns. These restorations were performed under a general anesthetic approximately 3 years prior to the initial orthodontic consultation. Assessment of the maxillary and mandibular arches revealed significant crowding in the canine regions bilaterally.

**FIGURE 1:**
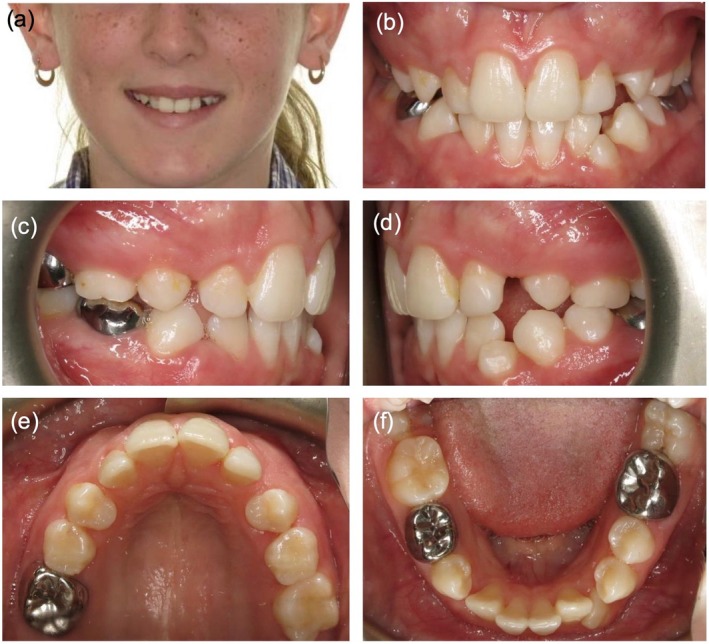
(a–f) Pre‐treatment photographs of a 12‐year‐old patient in the late mixed dentition. The bilateral molar relationship was Class I. The right maxillary first permanent molar (16), the mandibular left first permanent molar (36), and the mandibular right second primary molar (75) had been previously restored with stainless‐steel crowns. Assessment of the maxillary and mandibular arches revealed significant crowding in the canine regions bilaterally.

A panoramic radiograph (Figure 2a) revealed that previous endodontic procedures had been performed for the 16 and 36 (i.e., a pulpotomy and pulpectomy, respectively) prior to the placement of stainless‐steel crowns. It also appeared that the 17, 27, and 37 may not have sufficient space for complete eruption due to an apparent posterior arch length deficiency. The 38 and 48 appeared to have unfavorable crown angulations at this time. The 13, 23, 33, and 43 were also confirmed to be lacking in space for clinical eruption into satisfactory alignment.

**FIGURE 2:**
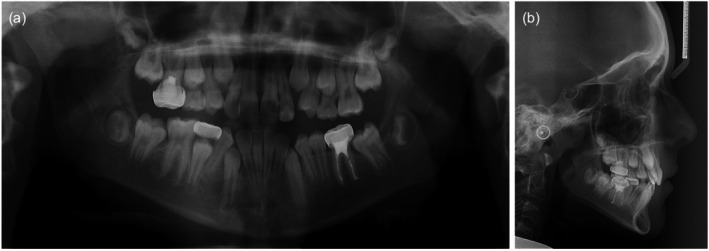
(a) The panoramic radiograph revealed that previous endodontic procedures had been performed for the 16 and 36 prior to the placement of stainless‐steel crowns. It also appeared that the 17, 27, and 37 may not have sufficient space for complete eruption due to an apparent posterior arch length deficiency. The 38 and 48 appeared to have unfavorable crown angulations at this time. The 13, 23, 33, and 43 were also confirmed to be lacking in space for clinical eruption into satisfactory alignment. (b) A lateral cephalograph revealed that the patient had a mild horizontal skeletal Class II relationship, a mesofacial vertical facial morphology, and normal angulation of the maxillary and mandibular incisors.

Routine pre‐treatment cephalometric assessment diagnosed a mild horizontal skeletal Class II relationship and normal mesofacial vertical skeletal morphology. The maxillary and mandibular incisors were well‐positioned, and the lip function was competent (Figure 2b). The relevant diagnostic findings are summarized in Table 1. Several potential treatment options were considered. The advantages and disadvantages of each treatment option were discussed (Table 2).

**TABLE 1:** Key diagnostic features.

Skeletal
Mild Class II skeletal base (Horizontal)
Mesofacial growth pattern (Vertical)
Normal facial symmetry (Transverse)
Dental
Class I molar relationship (Horizontal)
Normal overjet ~2.5 mm (Horizontal)
Normal anterior overbite (Vertical)
Mandibular dental midline located 1 mm to the left side of the maxillary dental midline and the facial midline (Transverse)
No posterior crossbites (Transverse)
Soft Tissues
Normal lip competency (Horizontal)
Normal gingival display on smiling (Vertical)
Normal chin point position (Transverse)
Other Diagnostic Features of Note
Moderate–severe crowding in both the maxillary and mandibular arches
Labial displacement of the maxillary and mandibular canine teeth
Likely future impaction of the maxillary and mandibular second and third permanent molars
Previous endodontic treatment (i.e., pulpotomy for the 16 and pulpectomy for the 36) with subsequent placement of stainless‐steel crowns
Third molar teeth present in the early stage of formation

**TABLE 2:** Relevant treatment options.

Treatment Option 1:	No active orthodontic treatment. Maintain the restored 16 and 36 and provide any future endodontic and prosthodontic treatment as required.
Advantages:	Given that the 16 and 36 were currently asymptomatic, this option does not commit the patient to any active intervention.
	Does not necessarily preclude future orthodontic treatment.
Disadvantages:	The compromised 16 and 36 are likely to progress through the restorative cycle throughout the patient's lifetime. Any additional treatment will have associated financial, biological, and opportunity costs for the patient.
	The 13, 23, 33, and 43 will likely erupt in the labial sulcus due to the significant intra‐arch crowding. The 37 is also more likely to become impacted, which may result in long‐term periodontal concerns and/or caries.
	The 18, 28, 38, and 48 are highly likely to become impacted in the long‐term.
	The criteria for normal occlusion will not be achieved with this option.
Treatment Option 2:	No active orthodontic treatment. Extract the restored 16 and 36 and allow for physiologic drift of the teeth in the first and third quadrants of the mouth.
Advantages:	Avoids the restorative cycle for the compromised 16, 36, and simultaneously permits resolution of the significant anterior and posterior intra‐arch crowding in the first and third quadrants of the mouth. This may permit improved alignment of the 13, 33, and 37.
	Does not necessarily preclude future orthodontic treatment.
Disadvantages:	Likely change in the maxillary and mandibular midline positions due to this asymmetric extraction pattern (although this can be corrected with future balancing extractions in the second and fourth quadrants and comprehensive orthodontic treatment). Residual extraction space and non‐ideal root parallelism may result, particularly in the third quadrant.
	The 23 and 43 are likely to erupt in the labial sulcus due to the unresolved significant intra‐arch crowding in the second and fourth quadrants.
	The 28 and 48 are highly likely to become impacted in the long‐term.
	The criteria for normal occlusion will not be achieved with this option.
Treatment Option 3:	Extraction of the 16, 26, 36, 46 in conjunction with comprehensive orthodontic treatment to close all extraction spaces.
Advantages:	Avoids the restorative cycle for the compromised 16, 36, and simultaneously permits resolution of the significant anterior and posterior intra‐arch crowding.
	Permits a symmetrical orthodontic treatment and a normal Class I post‐treatment occlusion.
	Provides the maximum potential for future eruption of the 18, 28, 38, 48 into functional occlusion.
Disadvantages:	Orthodontic treatment to close large residual extraction spaces is more challenging and likely increases the overall treatment duration.
	Requires access to orthodontic treatment, along with excellent patient compliance and motivation throughout such orthodontic treatment.
	Future successful eruption of the 18, 28, 38, 48 into functional occlusion cannot be guaranteed (periodic and long‐term reassessment is required).
Treatment Option 4:	Extraction of the 16, 25, 36, 45 in conjunction with comprehensive orthodontic treatment to close all extraction spaces.
Advantages:	Avoids the restorative cycle for the compromised 16, 36 and simultaneously permits resolution of the significant anterior and posterior intra‐arch crowding.
	Reduces the amount of extraction space closure required, which may reduce the overall treatment duration.
	Provides an increased potential for the eruption of the 18, 28, 38, 48 into functional occlusion.
Disadvantages:	Involves asymmetric orthodontic treatment due to the asymmetric extraction spaces. Asymmetric orthodontic treatment may increase the difficulty in providing ideal dental midline positions and will not permit symmetrical therapeutic molar relationships.
	Requires access to orthodontic treatment, along with excellent patient compliance and motivation throughout such orthodontic treatment.
	Future successful eruption of the 18, 28, 38, 48 into functional occlusion cannot be guaranteed, particularly in the second and fourth quadrants (as removal of the 25, 45 will not provide as much posterior arch space as removing the 16, 36).
	The criteria for normal occlusion will not be achieved with this option.
Treatment Option 5:	Extraction of the 14, 24, 35, 45 in conjunction with comprehensive orthodontic treatment to close all extraction spaces.
Advantages:	Provides the least complicated orthodontic treatment to resolve the significant intra‐arch crowding
	Permits a symmetrical orthodontic treatment and a normal Class I post‐treatment occlusion.
	Slightly increases the potential for eruption of the 18, 28, 38, 48 into functional occlusion.
Disadvantages:	Does not address the compromised 16, 36. The 16 and 36 are likely to progress through the restorative cycle throughout the patient's lifetime.
	Requires access to orthodontic treatment, along with excellent patient compliance and motivation throughout such orthodontic treatment.
	Future successful eruption of the 18, 28, 38, 48 into functional occlusion cannot be guaranteed (as removal of these premolar teeth will not provide as much posterior arch space as removing the first permanent molars).

Following an extensive discussion between the orthodontist, general dentist, and the patient's family, it was determined that removal of the maxillary and mandibular first permanent molars, followed by comprehensive orthodontic treatment, had the greatest potential to provide an ideal treatment outcome from both an orthodontic and long‐term restorative perspective. Although successful restorative treatment can certainly demonstrate good longevity, the relatively young age of the patient was a primary consideration, as further progression into the restorative cycle for the 16 and 36 would be expected over the patient's lifetime. The pre‐existing crowding would also become more evident as the patient matured; therefore, symmetrical extractions and subsequent orthodontic treatment appeared to be well justified.

## 3 Methods

The patient was referred for removal of the 16, 26, 36, 46 under a local anesthetic with the general dentist, and the process was uneventful. The patient exhibited good oral hygiene and was motivated to undergo orthodontic treatment, which commenced two months post‐extraction.

Maxillary and mandibular labial fixed appliances (Victory Series 0.022″ × 0.028″ slot, MBT prescription, 3 M Oral Care, MN, USA) were placed. A nickel‐titanium coil spring was placed on the archwires to open additional space for the labially erupting maxillary and mandibular permanent canines. The maxillary and mandibular second permanent molars were bonded to achieve their required mesial movement with an elastomeric chain.

At 12 months into active orthodontic treatment, the progress photographs revealed significant extraction space closure had been achieved, along with successful alignment of the maxillary and mandibular canine teeth (Figure 3).

**FIGURE 3:**
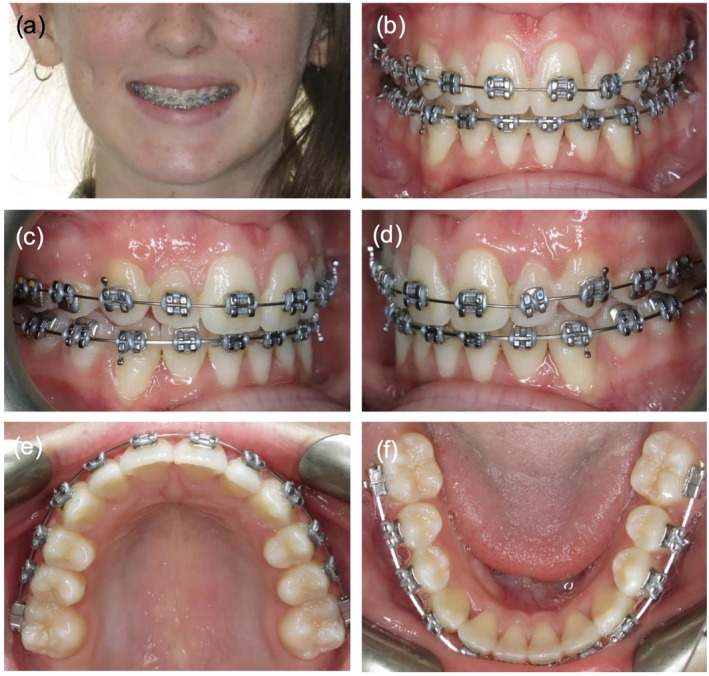
(a–f) Progress photographs taken 12 months into active orthodontic treatment revealed significant extraction space closure had been achieved, along with successful alignment of the maxillary and mandibular canine teeth.

A progress panoramic radiograph and lateral cephalograph were also obtained. The progress panoramic radiograph (Figure 4a) demonstrated favorable closure of the first permanent molar extraction spaces; however, the maxillary second molar roots were determined to have excessive distal root angulation. The maxillary and mandibular third molars showed evidence of further development; however, the 38 and 48 appeared to have an unfavorable crown angulation. The lateral cephalograph revealed that despite the extraction space closure, normal angulation of the maxillary and mandibular incisor teeth and a pleasing soft tissue profile had been maintained (Figure 4b).

**FIGURE 4:**
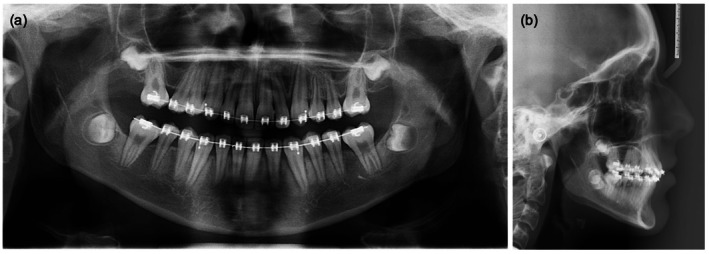
(a) A progress panoramic radiograph taken 14 months into active orthodontic treatment demonstrated favorable closure of the first permanent molar extraction spaces; however, the maxillary second molar roots were determined to have excessive distal root angulation. The maxillary and mandibular third molars showed evidence of further development; however, the 38 and 48 appeared to have an unfavorable crown angulation. (b) The lateral cephalograph, also taken 14 months into active orthodontic treatment, revealed that despite the extraction space closure, normal angulation of the maxillary and mandibular incisor teeth and a pleasing soft tissue profile had been maintained.

The fixed orthodontic appliances were removed after 21 months of active treatment. Stainless steel wire retainers were bonded to the maxillary and mandibular anterior teeth (Figure 5), with full‐coverage maxillary and mandibular vacuum‐formed retainers (Essix C+ material, Raintree Essix Inc., Zendura) also provided. Instructions were given for the vacuum‐formed retainers to be worn indefinitely.

**FIGURE 5:**
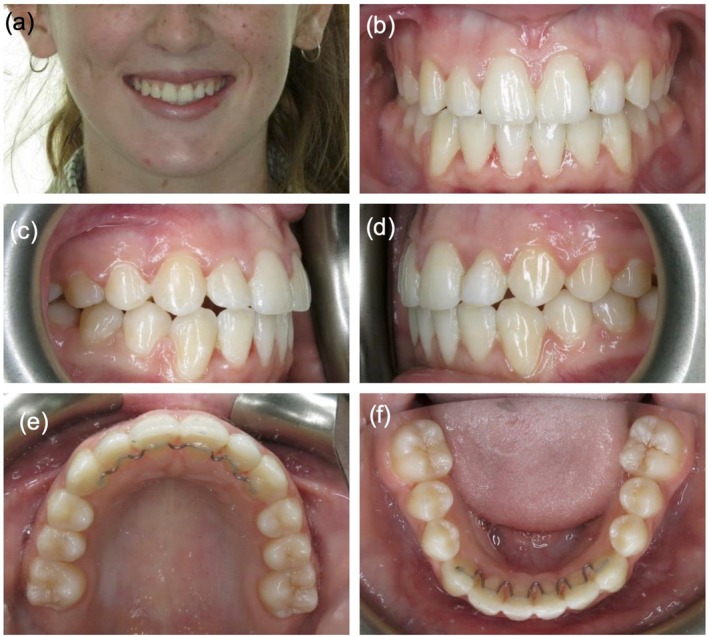
(a–f) Following 21 months of active orthodontic treatment, the fixed appliances were removed, with bonded retainers placed on the maxillary and mandibular anterior teeth.

The importance of indefinite retention with fixed and removable retainers was discussed in detail prior to commencing and reiterated prior to the completion of the active orthodontic treatment. Diligent wear of the maxillary and mandibular vacuum‐formed retainers was essential to avoid any significant over‐eruption and/or distal drift of the maxillary and mandibular second permanent molars, as such movements could potentially reduce the eruption potential of the developing third molars.

An updated panoramic radiograph (Figure 6a) assessed the overall root parallelism and the positions of the third molar teeth. The excessive distal root angulation of the 17 and 27 had been successfully corrected since the previous panoramic radiograph (taken at 14 months into active treatment), thus providing a more favorable eruption pathway for the 18 and 28. In contrast, the 38 and 48 appeared to have unfavorable crown angulation. It was advised that long‐term monitoring would be required for the unerupted third molars and that their successful eruption into functional occlusion could not be guaranteed.

**FIGURE 6:**
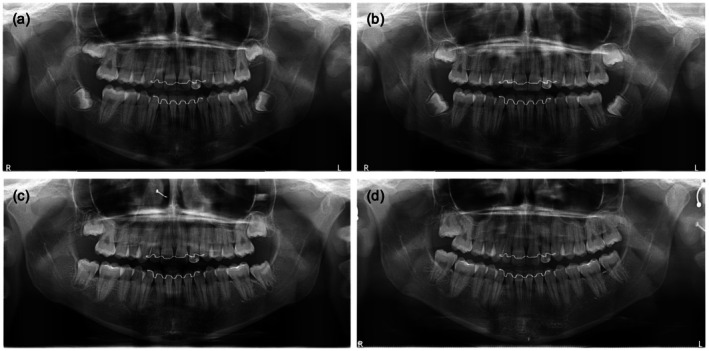
(a) An updated panoramic radiograph was taken at the completion of the active orthodontic treatment to review the overall root parallelism and the positions of the third molar teeth. The excessive distal root angulation of the 17 and 27 had been successfully corrected since the previous panoramic radiograph. (b–d) Post‐treatment panoramic radiographs were taken at 1.5 years, 4 years, and 6.5 years, respectively.

Retention reviews with sequential panoramic radiographs were performed at 1.5 years (Figure 6b), 4 years (Figure 6c), and 6.5 years (Figure 6d) following the completion of the original comprehensive orthodontic treatment. The patient was 18 years and 21 years of age at the 4‐year and 6.5‐year post‐treatment reviews, respectively (Figure 7). It was noted that the 28, 38, and 48 were erupting favorably; however, the 18 had partially erupted and was impacted against the distal aspect of the 17.

**FIGURE 7:**
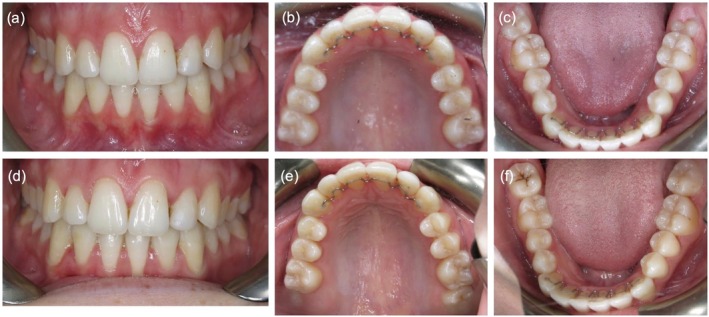
(a–c) The patient presented for a clinical review at 18 years of age, which was 4 years after the completion of the original comprehensive orthodontic treatment. The 18 and 28 remained unerupted; however, the 38 and 48 were partially erupted. (d–f) The patient was reviewed again at 6.5 years post‐treatment, with the patient 21 years of age at that time. It was noted that the 28, 38, and 48 were erupting favorably; however, the 18 had partially erupted and was impacted against the distal aspect of the 17.

With informed consent, a decision was made to place sectional fixed appliances in the posterior segment of the first quadrant to distalize and align the impacted 18 (Figure 8). A self‐ligating molar tube Damon Q (Ormco, CA, USA) was placed on the 17 to permit easy placement of a compressed active open coil spring between the 17 and 18 to effectively distalize the 18. The 18 was disimpacted with a combination of the sectional 0.014″ nickel‐titanium archwire and the active coil spring. The sectional fixed appliances were removed after a treatment duration of 3 months. Maxillary and mandibular vacuum‐formed Essix removable retainers were re‐made to cover the third molar teeth.

**FIGURE 8:**
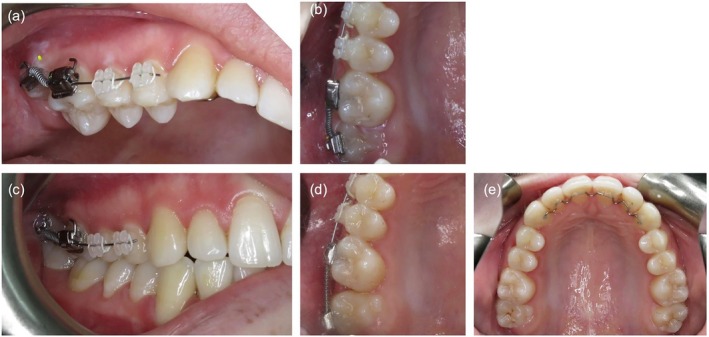
(a,b) Sectional fixed appliances were placed in the posterior segment of the first quadrant to distalize and align the impacted 18. (c,d) Photographs taken following 2 months of sectional fixed appliance treatment demonstrate the efficient and effective distal and occlusal movement of the previously impacted 18. (e) The sectional fixed appliances were removed following 3 months of active treatment.

## 4 Conclusions and Results

The patient was reviewed 2 years after completion of the sectional orthodontic treatment to disimpact the 18 (Figure 9). Minor gingival recession associated with tooth 31 was noted, which had become periodically symptomatic. The flossable lower fixed retainer was removed to determine if any wire distortion had contributed to the gingival recession. Inadvertent tooth movement may result from unforeseen retainer wire deformation and has been previously reported in the literature [11]. An intraoral scan was acquired to assess the 31 and to quantitatively confirm the favorable posterior occlusal interdigitation (Figure 10). Although the 31 root was not unduly labially positioned, the recession issue may have resulted from the narrow anatomical labio‐lingual alveolar ridge width. The lower fixed retainer was replaced with a simpler 0.5 mm stainless‐steel wire design. A panoramic radiograph was taken to assess alignment of the 18, along with the generalized alveolar bone height (Figure 11). The patient was referred to a periodontist to manage the 31 recession.

**FIGURE 9:**
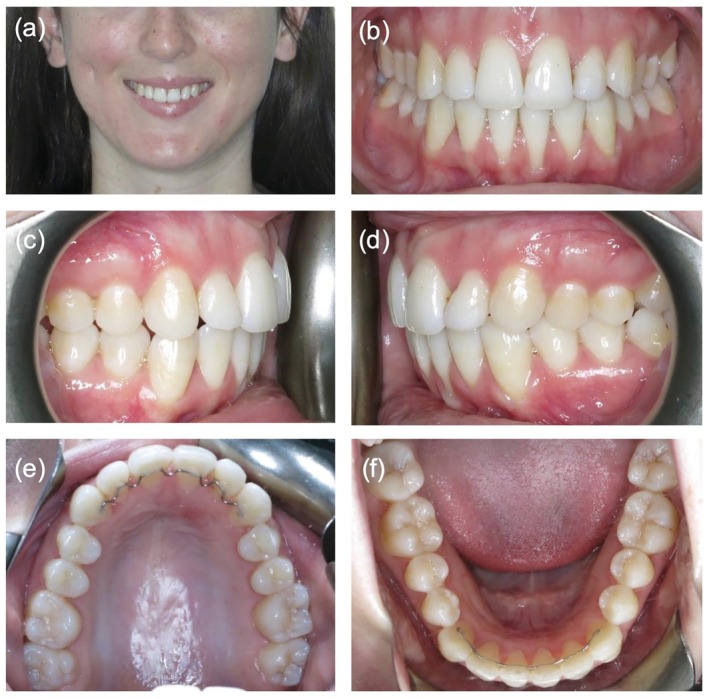
(a–f) The patient was reviewed 2 years after completion of the sectional orthodontic treatment to disimpact the 18, and was noted to be 23 years of age at this time. The patient had noted some gingival recession associated with the 31, which had become periodically symptomatic.

**FIGURE 10:**
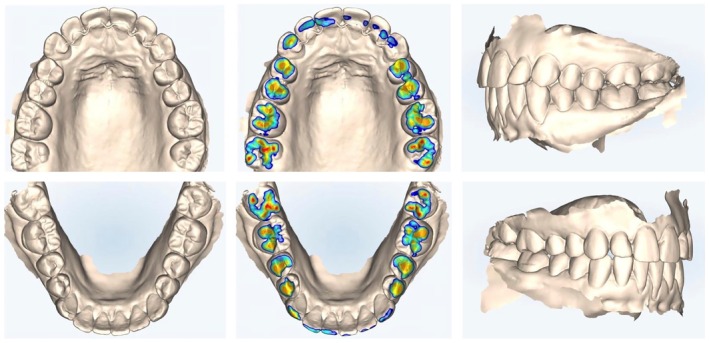
An intraoral digital scan was acquired to more closely assess the 31 and quantitatively confirmed the favorable occlusal interdigitation of the maxillary and mandibular posterior teeth, including the third molars.

**FIGURE 11:**
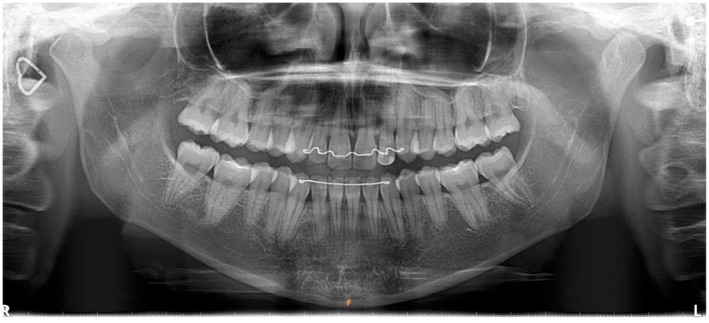
A final panoramic radiograph was obtained, which was used to assess the alignment of the 18, along with the generalized alveolar bone height.

## 5 Discussion

The presence of compromised first permanent molars is a relatively common clinical scenario in adolescent patients [1, 2, 3, 4]. Should the removal of compromised first permanent molars be recommended, comprehensive orthodontic assessment and treatment are generally required to provide ideal treatment outcomes [6, 7].

Should a decision be made to retain compromised permanent teeth, careful consideration must be given to the type and expected longevity of the restoration provided. Fissure sealants, glass ionomer cement, composite, and stainless‐steel crowns are commonly used restorative materials for compromised teeth in a pediatric patient. The choice of restorative material is subject to the enamel quality and structural integrity of the tooth, patient compliance, and the clinician's preference and experience.

Stainless‐steel crowns may be used to restore the primary and permanent dentition, particularly for adolescent patients. Stainless‐steel crowns offer several advantages compared to a composite resin restoration, which include the relative ease of placement and enhanced durability. The margins of a stainless‐steel crown may initially be located subgingivally if the crown is placed on a tooth during its early eruptive phase. With further clinical eruption and time, the crown margins may become exposed, and occlusal perforation may also eventuate. Despite the reduced technique sensitivity compared to other restorations, a stainless‐steel crown may still have the potential for a poor fit. A poorly adapted stainless‐steel crown and/or excess cement may result in periodontal concerns (e.g., gingivitis, localized bone loss) and impaction of the adjacent erupting tooth, which was noted in this case report.

A retrospective study evaluated the longevity of stainless‐steel crowns on permanent teeth and reported an average longevity of 43 months [12, 13]. Another retrospective study investigated the survival probability of stainless‐steel crowns placed on first permanent molars following vital pulp therapy in adolescent patients. The study concluded that the cumulative survival probability of stainless‐steel crowns decreased over time, with a 39.6% survival rate after 60 months in situ [14]. Eventual replacement of a stainless‐steel crown with an indirectly fabricated full coverage crown is the likely next step in the restorative cycle for the compromised molars. Unfavorable long‐term progression into the restorative cycle may require additional endodontic treatment, post and core preparation, crown replacement, and, in the event of catastrophic tooth or restorative failure, extraction and/or restorative implant placement.

Although a healthy tooth without any prior restoration is ideal, the need for endodontic treatment does not necessarily lead to a poor long‐term prognosis. It has been established that the preservation of intact coronal and radicular tooth structure, particularly the cervical tissue to create a ferrule effect, is important for providing optimal restorative biomechanics [15, 16]. A ferrule has been defined as an encircling band of cast metal around the coronal surface of a tooth. A restoration's ferrule effect is defined as a band that surrounds the external dimension of the residual tooth structure [17].

Adolescent patients with one or more compromised first permanent molars may have immature and unerupted third molar teeth. When compromised, the first permanent molars are removed, and the developing third molar teeth have a higher likelihood of spontaneous eruption due to the additional posterior arch space [9, 18]. Orthodontic treatment can provide effective extraction space closure and provide favorable root parallelism for the relocated second permanent molars to improve the probability of third molar eruption. Unfortunately, the provision of orthodontic treatment requires significant patient motivation and compliance and does not guarantee future third molar eruption and functional occlusion.

It is also pertinent to discuss an option that involves the removal of compromised first permanent molars without active orthodontic treatment, as not every patient will have access to orthodontic treatment, nor have the required motivation and compliance. Historical studies [6, 8] have proposed that the developmental stage of the second permanent molar is a critical factor for promoting spontaneous closure of the first permanent molar extraction space. Despite these postulations, the response of the second permanent molar is subject to significant variation, particularly in the mandibular arch [19]. In the maxillary arch, complete space closure was reported in 92% of first permanent molar extraction cases, irrespective of the developmental stage of the maxillary second permanent molar. In contrast, despite extracting the mandibular first permanent molar at the previously reported ideal time (i.e., the bifurcation developmental stage of the second permanent molar), only 66% of these cases demonstrated complete space closure [19].

The third molars have the highest probability of impaction, with a prevalence ranging from 9.5% to 39% among various populations [20]. Insufficient retromolar space [21, 22, 23, 24], deficient mandibular growth [23], a distal eruption path [21], a vertical condylar growth direction [23], an increased mesiodistal crown dimension [21], and late maturation [25] have been cited as potential reasons for third molar impaction.

Although previous studies [20, 21, 22, 23, 24, 26, 27, 28], have attempted to determine the prognosis of third molar eruption, it has also been demonstrated that neither orthodontists nor maxillofacial surgeons can reliably predict the spontaneous eruption of the mandibular third molars [28].

This case report showcases the unpredictability of the third molars. The orthodontist assumed that the mandibular third molars would not erupt spontaneously after assessing their positions immediately post‐treatment and at 1.5 years post‐treatment (Figure 6). The orthodontist also incorrectly expected that both maxillary third molars would erupt more favorably. This highlights the need for long‐term monitoring of first permanent molar extraction cases, particularly when there is an expectation for future third molar eruption.

Fortunately, the patient was able to present for multiple review appointments and was willing to undergo subsequent sectional fixed appliance treatment to distalize and align the impacted 18. In general, sectional fixed appliance treatment is less visible and is easier to maintain with good oral hygiene, thus improving overall patient acceptance. Use of a self‐ligating molar tube was particularly useful for the placement of the active compressed open coil spring, as this avoided the need to thread the archwire through the molar tube on the 17. By design, self‐ligating brackets and molar tubes have a clip or slide gate mechanism, which permits an efficient and secure ligation method. Contemporary self‐ligating appliance designs draw inspiration from the Russell edgewise attachment, developed in the 1930s [29, 30]. This self‐ligating bracket prototype was introduced to reduce orthodontic ligation times and improve operator efficiency [31].

Achieving a normal occlusion is a central aim of orthodontic treatment. The six keys of normal occlusion were originally described by Andrews [32], with respect to the following criteria: Class I molar relationship, appropriate mesiodistal crown divergence, correct labiolingual and buccolingual crown angulation, no significant rotations, good proximal contact points, and a relatively flat curve of Spee. However, it is important to note that these objective occlusal keys are static parameters and primarily relate to the crowns of the teeth [33] Such morphologic criteria must also be accompanied by periodontally healthy tissues and a well‐functioning stomatognathic system to represent a successful treatment outcome.

A harmonious and complete dental arch occlusion was achieved for this patient. However, if an alternate scenario had arisen where the third molars did not spontaneously erupt and/or could not be orthodontically aligned into more ideal positions (i.e., therefore necessitating their removal), a shortened dental arch may still be considered functionally acceptable [34, 35]. Although a complete dentition is not a rigorous pre‐requisite for an adolescent patient to enjoy the lifetime benefits of a healthy and functional dentition, the therapeutic occlusion for this patient represents the best possible treatment outcome.

This case report demonstrates the long‐term management for an adolescent patient presenting with two compromised first permanent molars and how the initial comprehensive orthodontic treatment and a later revision with sectional orthodontic mechanics provided an ideal outcome for this patient. It is acknowledged that the apparent success for this individual patient may not necessarily be reproducible under different scenarios and requires access to orthodontic treatment, along with excellent patient compliance and motivation.

The presence of retro‐molar space provided by the removal of compromised first permanent molars and subsequent orthodontic mesialization of the second permanent molar teeth can increase the likelihood of successful third molar eruption. Although third molars are notorious for having a high probability of impaction and unfavorable changes in angulation, spontaneous eruption of these oft‐maligned teeth into functional positions can greatly enhance the overall treatment outcome. The inherent unpredictability of spontaneous third molar eruption emphasizes the value of long‐term follow‐up for patients who have undergone the extraction of one or more first permanent molars.

## Author Contributions

**Desmond Cheer‐Vern Ong:** conceptualization and treating clinician. **Yen Tran:** conceptualization.

## Funding

The authors have nothing to report.

## Consent

Written informed consent was obtained for the publication of this case report.

## Conflicts of Interest

The authors declare no conflicts of interest.

## Data Availability

The data for this case report is available upon request.
